# The need for speed: global optic flow speed influences steering

**DOI:** 10.1098/rsos.160096

**Published:** 2016-05-04

**Authors:** Georgios K. Kountouriotis, Callum D. Mole, Natasha Merat, Richard M. Wilkie

**Affiliations:** 1 Department of Psychology, Manchester Metropolitan University, Manchester M15 6GX, UK; 2 School of Psychology, University of Leeds, Leeds LS2 9JT, UK; 3 Institute for Transport Studies, University of Leeds, Leeds LS2 9JT, UK

**Keywords:** locomotion, steering, optic flow, driving, paths, asymmetry

## Abstract

How do animals follow demarcated paths? Different species are sensitive to optic flow and one control solution is to maintain the balance of flow symmetry across visual fields; however, it is unclear whether animals are sensitive to changes in asymmetries when steering along curved paths. Flow asymmetries can alter the global properties of flow (i.e. flow speed) which may also influence steering control. We tested humans steering curved paths in a virtual environment. The scene was manipulated so that the ground plane to either side of the demarcated path produced larger or smaller asymmetries in optic flow. Independent of asymmetries and the locomotor speed, the scene properties were altered to produce either faster or slower globally averaged flow speeds. Results showed that rather than being influenced by changes in flow asymmetry, steering responded to global flow speed. We conclude that the human brain performs global averaging of flow speed from across the scene and uses this signal as an input for steering control. This finding is surprising since the demarcated path provided sufficient information to steer, whereas global flow speed (by itself) did not. To explain these findings, existing models of steering must be modified to include a new perceptual variable: namely global optic flow speed.

## 1. Introduction

**Figure 1.**
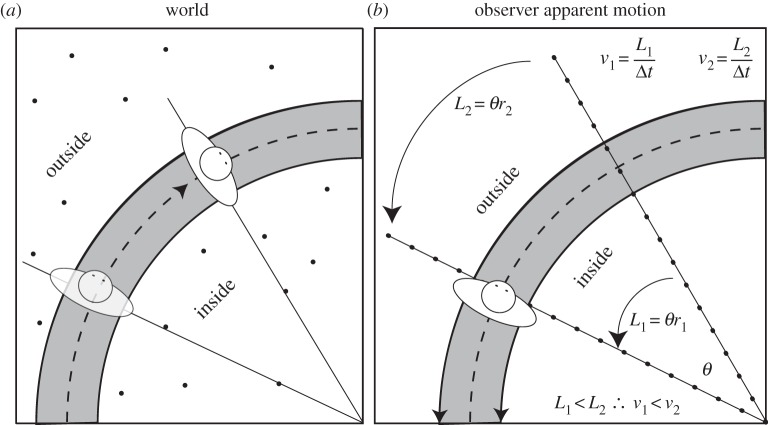
(*a*) Observer moving through a world with a textured surface. (*b*) Apparent motion resulting from (*a*). During curvilinear locomotion optic flow, vectors have different velocities across the scene depending on their proximity to the centre of rotation. Here, a constant curvature path is shown, but the same principle applies to all nonlinear paths. The apparent motion of flow elements near the centre of the rotation (*L*_1_) will be slower than that of flow elements further away from the centre of rotation (*L*_2_). In order for the observer to extract locomotor speed from optic flow, they would have to sample flow vectors from across the scene and average them.

**Figure 2.**
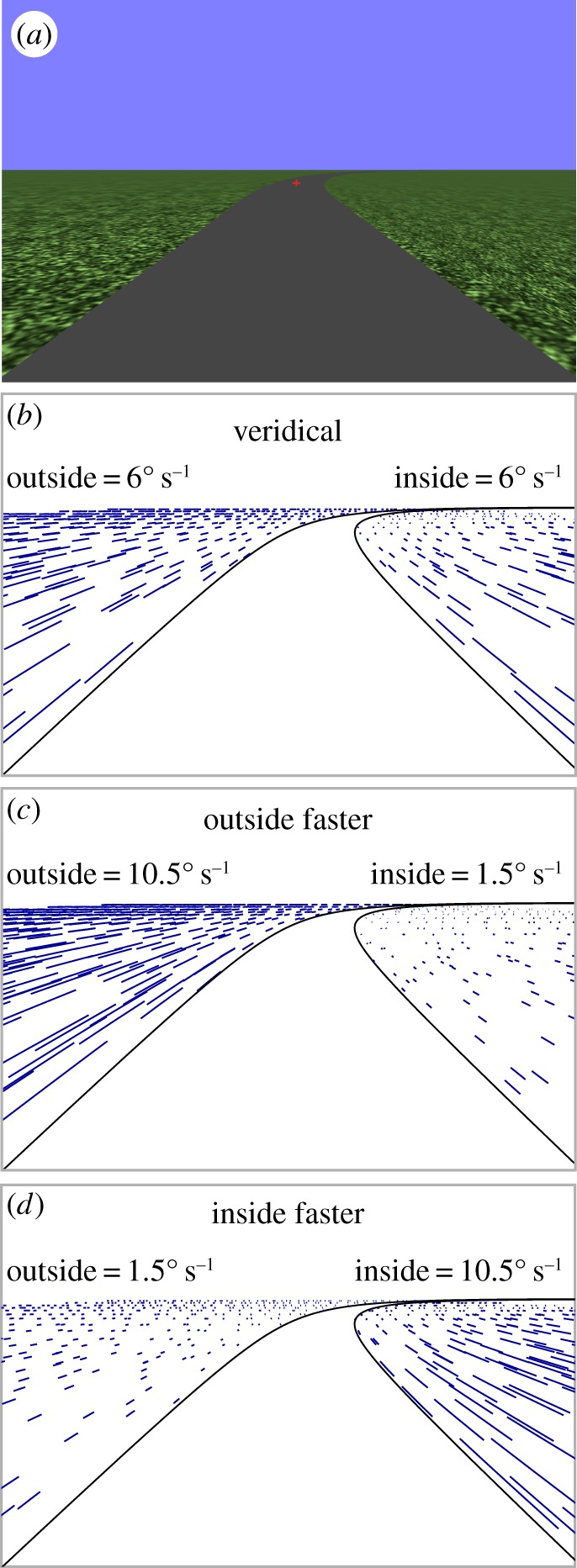
(*a*) Example display containing a single ground surface made up of two textured regions (inside and outside of the path; figure 1) as well as the non-textured path. A fixation cross was displayed at the road centre at a constant 16.1 m (approx. 1.2 s) ahead. (*b*) Veridical flow conditions matched flow speeds to the locomotor speed and caused ‘natural’ asymmetries. (*c*) Outside faster conditions enhanced asymmetries by increasing the speed of the outside region, and reducing the speed of the inside region. (*d*) Inside faster conditions increased the speed of the inside region, and reduced the speed of the outside region (this is the slow speed, large asymmetry condition listed in table 1).

Animals routinely move through the world by following demarcated paths, trails or runways [1–3]. These paths specify the locomotor requirements as well as providing the information necessary for generating successful trajectories (i.e. an immediate error correction signal from a near point and a prospective steering control signal from a far point [4]). However, an animal travelling across a ground surface also experiences the apparent perceptual motion of texture elements (often referred to as optic flow [5]). A wide variety of species are sensitive to optic flow during locomotion, including bees [6,7], flies [8], birds [9], desert ants [10] and humans [11,12]. Experiments conducted on humans travelling at walking speeds down a straight corridor [13–17] have shown that an asymmetric flow field (created by moving one corridor wall) can cause participants to adopt a trajectory closer to the slower moving wall. This finding has been explained in terms of humans attempting to optically equalize the differences in the flow vector speeds between the left and right visual fields. A similar equalization response has been observed in bees [7], but in humans the effect of asymmetric flow often disappears when additional visual information (akin to a demarcated path) is added to the environment [13]. Figure 1 demonstrates how, for a circular trajectory along a curvilinear path, flow vectors from a textured ground vary in speed across the scene depending on their proximity to the centre of the circle: the nearer to the centre, the slower the flow vector that is formed [18,19]. The pattern of these ‘natural’ asymmetries could be informative for steering, and it has been recently demonstrated that altering the natural flow asymmetries can influence steering along curvilinear trajectories despite the presence of a demarcated path [20]. While this work highlights that the signals from the demarcated path and flow both seem to influence steering, the precise way in which flow is used remains unclear. There are suggestions that the visual strategies employed by drivers (i.e. where they look and when) are an important component of steering [21]; however, the literature has not considered the relative contribution of flow asymmetries and global flow speed when steering down demarcated paths. Local flow speeds have been raised as a potential control variable for detecting differences in path curvature [22] but only in the absence of a demarcated path. Confusingly, the term ‘path’ can be used to describe a visible demarcated path (with distinct visible edges providing information even for a non-moving animal), but it can also refer to the future location of the animal based on the current rate of steering as perceived purely from optic flow (i.e. ‘future path’ [23]). In this paper, any reference to path solely relates to the former definition of a demarcated path with visible edges (figures 1 and 2).

## 2. Material and methods

To test the relative influence of path information, flow asymmetry and global flow speed, a virtual reality environment was created that rendered a series of trials containing constant curvature (3 m wide) bending paths with a separate textured ground region either side (labelled ‘inside’ or ‘outside’) that could be independently rotated (figure 2; also see [20]). The path is naturally located on the ground, so generating flow from regions either side of the path meant that when looking at the fixation point on the path both sources (flow and path) would be available. Critically, this also meant that we were able to manipulate flow speeds independent of locomotor speeds and path information, keeping all other variables constant, and without causing obvious conflicts whereby the texture elements appeared to move underneath/across the path (the path itself was left untextured as per [20]). Nineteen human participants used a wheel to steer down each bending path while trying to maintain a central position. Locomotor speed relative to the path edges was kept constant at 13.8 m s^−1^ (13.2° s^−1^ on a bend with a 60 m radius). The inside and outside ground regions were rotated around the centre of the circle describing the constant curvature bends to create conditions whereby: (i) one region was always moving faster or slower than the other, (ii) the difference between the rotation speeds of the inside and outside region was either artificially small or large and (iii) the average speed of the inside and outside was either slower than actual travelling speed (6° s^−1^), equal to actual travelling speed (13.2° s^−1^) or faster than travelling speed (26.4° s^−1^). Table 1 gives the rotation values used when the inside region rotated faster than the outside region. Conditions where both regions were rotated by the same amount at slow, medium or fast speeds were also tested. The ‘natural asymmetry medium speed’ condition created flow that was veridical and matched the locomotor speed (as specified by movement of the observer relative to the visible path edges).

**Table 1.** Rotation speeds for the ‘inside faster’ conditions. The speeds used in the ‘outside faster’ conditions were identical but switched from outside to inside (and vice versa). SMAS = smaller asymmetry, LGAS = larger asymmetry. Speeds are shown in degrees per second.

	slow speed (6° s^−1^)		medium speed (13.2° s^−1^)		fast speed (26.4° s^−1^)	
region	SMAS	LGAS	SMAS	LGAS	SMAS	LGAS
outside	4.5	1.5	9.9	3.3	19.8	6.6
inside	7.5	10.5	16.5	23.1	33.0	46.2

For each trial, we calculated steering bias, taking the position of the participant in the world for each frame (60 frames s^–1^) and finding the closest distance to the (invisible) centreline of the demarcated path. Steering bias measured whether participants spent most of the trial in the centre of the path (zero bias), or whether steering caused drift towards the inside path-edge (positive bias) or towards the outside path-edge (negative bias). Each experimental condition was repeated six times (with conditions randomly interleaved) and a mean was taken to provide an estimate of bias for each condition for each person. Statistical evaluation of steering bias was then carried out using a repeated-measures ANOVA using SPSS 20.

## 3. Results and discussion

**Figure 3.**
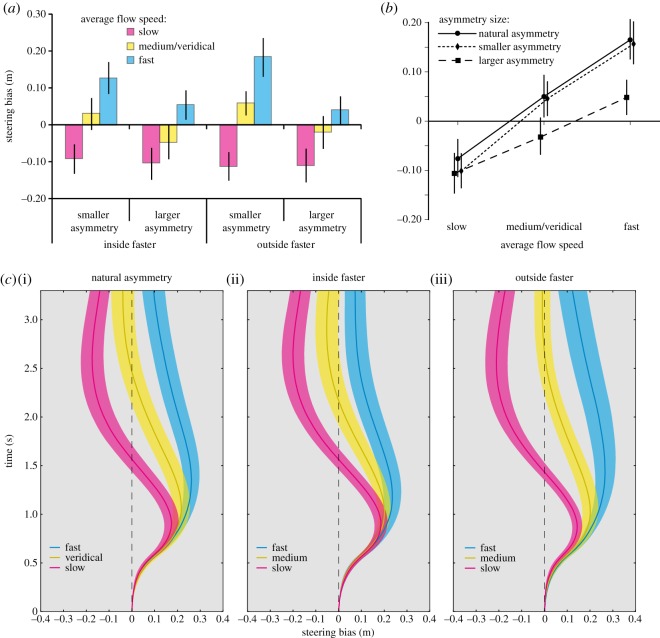
(*a*) Steering bias in asymmetric conditions. Participants were influenced by the average flow speed in a manner consistent with sampling flow vectors from across the scene and then averaging them to derive global flow speed. Steering patterns were not consistent with the flow equalization strategy (where it would be expected to see understeering in the ‘inside faster’ conditions and oversteering in the ‘outside faster’ conditions). (*b*) The interaction between ‘asymmetry size’ and ‘average speed’ in steering bias, with the equivalent data for the symmetric conditions plotted. Participants were influenced more by the average speed when there was a small difference between the rotation speeds of the two regions, and steering biases were of similar magnitude to conditions that had no asymmetries. (*c*) Average steering biases over time (lighter coloured regions indicate s.e.m.) for all flow conditions. The biases observed are in line with participants using speed cues from global flow to influence their steering responses. The patterns of steering bias unfolding over time are similar for all asymmetry conditions irrespective of which region was rotated. All bars = s.e.m.

If participants attempted to steer in a way that reduced asymmetries and made the flow vectors either side of the path more equal, then we would expect: (i) trajectories to be biased towards the slower moving region, and (ii) the magnitude of steering biases to reflect the difference between the speeds of the two regions (i.e. larger biases to be caused by larger asymmetries). Our results (figure 3*a*) do not follow this pattern; instead, what drives the pattern of results is the *global flow speed* averaged across both regions. Participants systematically steered towards the outside of the bend when the average flow speed was slow and towards the inside of the bend when average flow speed was fast. This effect was independent of which region was moving faster or slower, and also regardless of the asymmetry magnitude.

Statistical analyses support our observations: a 2 (faster region) × 3 (average speed) × 2 (flow asymmetry size) repeated-measures ANOVA revealed no effect of whether the inside or the outside was the faster region (*F*_1, 18_ = 0.61, *p* = 0.445) and no interaction between faster region and flow asymmetry size (*F*_1, 18_ = 0.58, *p* = 0.457). Instead, an effect of average speed (*F*_2, 36_ = 35.37, *p* < 0.001, $\eta_{p}^{2}=0.66$), flow asymmetry size (*F*_1, 18_ = 18.82, *p* < 0.001, $\eta_{p}^{2}=0.51$) and an interaction between average speed and flow asymmetry size (*F*_2, 36_ = 5.81, *p* = 0.006, $\eta_{p}^{2}=0.24$) were found. The interaction was caused by participants exhibiting greater biases when flow asymmetry size was *smaller* (figure 3*b*), which is the opposite of what would be expected if the asymmetries were directly influencing steering behaviours. Rather it appears that greater asymmetries reduced the extent to which participants relied upon optic flow information to inform their steering. This is consistent with studies showing flexible weighted combination of information sources based on the variability of the information [24,25].

### 3.1. Natural asymmetry conditions

When both regions of the flow field were moved together in the same direction, slower (6° s^−1^) or faster (26.4° s^−1^) than actual travelling speeds, systematic changes in steering were also observed. There was an effect of global flow speed across these conditions (*F*_1.29, 23.04_ = 25.08, *p* < 0.001, $\eta_{p}^{2}=0.58$) as shown in figure 3*c*(i). Fisher's least significant difference comparisons indicated that all flow conditions differed from each other at the *p* < 0.05 level.

Taken together these findings provide compelling evidence that flow speed averaging (rather than a flow speed equalization) is used by humans when steering curvilinear trajectories along demarcated paths. In asymmetric flow conditions, steering was not influenced by which region moved slower or faster. Instead steering was influenced by the global flow speed, with systematic changes in steering across different flow speeds irrespective of the direction of flow speed asymmetries. It seems then, that there are differences between how humans and bees use optic flow speed. Whereas the bee steers by responding to asymmetries in flow speed, the human steers by responding to the global magnitude of flow speed. There are, however, still other remarkable parallels between bees and humans. Neither species relies upon a single coarse optic flow signal for controlling actions, rather, it seems that these animals make extensive and subtle use of a variety of cues that are present and available within the optic flow field [6].

One of the most interesting aspects of the present study is that global flow speed by itself does not specify the current or future steering requirement for the human (unlike other properties such as flow direction [12,25–28]), and so this input is not usually included in conventional steering models (though see [22]). While it seems most likely that flow speed interacts with demarcated path information (which does specify both the immediate and future steering requirements) further investigations are needed to establish the extent and nature of the interaction with established perceptual components of steering [4,27,29]. It should also be highlighted that the flow conditions examined here were purposely limited to a single surface: the ground. Self-motion across a textured ground is a common locomotor scenario, and environments that do not involve some form of ground texture are exceptional. Real-world conditions, however, will produce a variety of global flow signals, and in some cases stronger optic flow signals will be generated during self-motion (e.g. when additional textured surfaces are present such as walls, or trees and hedges, etc.) and richer flow fields should usually be expected to have a *greater* influence over steering (e.g. [12]). However, some of these surfaces will move independently of the observer (e.g. clouds and rivers) which could alter global flow speed estimates. Further work is needed to determine the extent to which global flow speeds combined across multiple surfaces influence steering along demarcated paths.

## 4. Conclusion

This study provides strong evidence that global flow speed from a ground plane has a profound effect on the trajectories humans take when steering along curvilinear demarcated paths, despite the continuous presence of veridical trajectory information from the visible path edges. This finding poses a problem for many models of steering control, which do not include flow speed as an explicit perceptual input. While asymmetric flow-speed conditions of the type simulated in this study are unlikely to occur naturally, the speed information from the global flow field can vary considerably across different real-world environments. Conditions where the quality of flow is degraded (e.g. the presence of fog or driving at night) can reduce the perceived locomotor speed [30,31], whereas increases in flow quality/quantity (e.g. driving with a seated position close to the ground along narrow country lanes) would increase perceived speed. Our findings indicate that such conditions could cause systematic steering errors even when there are clear visual markings for the position in lane and future steering requirements.

## Data accessibility

Our data can be accessed through figshare from http://dx.doi.org/10.6084/m9.figshare.3153217. Data also available from Dryad Digital Repository: http://dx.doi.org/10.5061/dryad.bb502.
